# Puerpal Convulsions

**Published:** 1893-04

**Authors:** J. E. Jameson

**Affiliations:** Jamaica Plain, Boston, Mass.


### PUERPERAL CONVULSIONS.

R. E. Jameson, M. D., Jamaica Plain, Boston, Mass.

Mrs. A., aged thirty-two, of a decidedly psoric constitution. Mother at climacteric season for four years and died of insanity. Her aunt, sister to her mother, died of puerperal convulsions at thirty-two. When eighteen was thrown from a carriage and spine injured. Suffered for several years with uterine disease, and received all kinds of local treatment at the hands of so-called homœopathic physicians. In January, 1882, gave birth to a daughter, and was in poor health for several years. Between the birth of her daughter and 1890, when I first became acquainted with her, had two miscarriages. In 1890 became pregnant again and called me in to relieve her of various symptoms incident to her condition, and prevent if possible another miscarriage. She had a good deal of leucorrhœa, constipation, headaches, and indigestion, with vomiting. Her headache and the gastric symptoms were aggravated by motion, and I gave her Bryonia$^{cm}$, which relieved all the symptoms but the leucorrhœa. For that I gave her Puls. and Sepia, which helped it very much but did not entirely cure it. She went to term and was delivered of a girl, that lived only twenty-four hours. She became pregnant again, as she was very anxious to have children, and had a much more comfortable time than at any previous pregnancy. April 4th last I was called about half-past twelve A. M. As she had previously had tedious labors her husband, who summoned me, did not hurry very much, and when I arrived I was very much surprised on being told that the baby had arrived. She had had a very easy labor, and was feeling very happy over the advent of the boy. I visited her again at half-past nine and found her comfortable with the exception of headache, which she said was not very bad and which did not alarm me, as she was so subject to headache. I left a remedy for it, however. At eleven o'clock I called at my house and was told by my wife that I was wanted at once at Mrs. A.'s, that she was in convulsions. I drove at once to her and found her in a violent spasm, the fourth that she had had. Her face was of a bluish color, there were twitchings of the muscles, especially of the face and eyelids; with great restlessness, tossing from one side of the bed to the other, with constant delirium. I gave her Hyoscyamus. In two hours there was apparently a little improvement in that she became conscious, and said she felt very uncomfortable and wanted to pass water. The improvement did not last, however, and I sent for Dr. Wesselhœft. The symptoms changed, there were now stupor, stertorous breathing, rigidity of limbs, head turned to right, eyes turned up, pupils dilated, and nearly insensible to light; pulse rapid; throws arms out at right angles with body, frothing at mouth, face livid and bloated, tongue swollen and abdomen very much distended and tympanitic. Hot sweat. I gave Opium, which was approved by Dr. Wesselhœft. Dr. Kimball came out at night, and remained till morning. We went over the symptoms together very carefully, and could find nothing better indicated than the Opium. In the night, however, the symptoms changed again. The stertor gave place to a constant moaning between the spasms. The spasm began in the right eyelid, and the right side was convulsed, while the left was motionless, as though paralyzed. The hot sweat continued, and there was rolling of the head on the pillow. Dr. Kimball gave Bell.$^{cm}$. The spasms were not quite so frequent, and from 5.30 to 8.30 she had none; between that and 11.30 she had five. I repeated the Bell. at 8.30 and at 10.30. At 11.30 she had the last one. Then respiration ceased and the pulse could scarcely be felt, and I thought she was gone. She remained unconscious till Wednesday night, about thirty hours after the convulsion ceased, when a violent delirium set in, and it was difficult to keep her in bed. Her screams could be heard over the neighborhood. I gave Coffea, Hyoscyamus, and Stramonium, as they seemed indicated. On Saturday her husband said her mental condition seemed like anger, real ugliness. I did not wonder that he thought so, for she struck him several times in the face. I asked him if she had shown any such disposition during her pregnancy or during labor. I had never seen anything of it, and had not been told of any such symptoms. He answered, " Oh! yes." The little daughter came to him one day and wanted him to take her somewhere because mamma treated her so, and sometimes it seemed impossible to do anything to please her. Saturday night or Sunday morning I gave her Chamomilla. Sunday P. M. I called, and found her in a quiet sleep. In the evening I called again, and she was still sleeping, though she had awakened once or twice and taken nourishment. From that time she improved, and is now perfectly well.

### Mastitis.

Mrs. F., about two weeks after confinement, called me in to see her breast, which was somewhat inflamed, but not very painful. It was on the right side, and the inflammation was of a pale red. I gave Bryonia, and in a few days called again and found it worse; the inflammation had increased and extended in area. She said the pains were pricking in character. It looked very much as though an abscess was inevitable. But she had another symptom. She said she was lame when she sat down, and it hurt her to rise from her seat. She was also constipated, had no stool without an enema. I gave her Lycopodium$^{cm}$, two doses. The abscess was averted, the constipation relieved, and the ischiatic lameness disappeared with the rest.

### Discussion.

Dr. Plummer—I had a case of convulsions strikingly similar to the one Dr. Jameson has reported, and occurred at very nearly the same time. In my case as in his, Hyoscyamus seemed indicated, yet did not help; but Opium gave marked relief.

It was, however, insufficient, and had to be followed by Belladonna.

My patient had paralysis of the right side following the attack, which cleared up in the course of ten days or two weeks under Phosphorus, and at the end of four weeks she was entirely cured.

Dr. Carr—Does the doctor recall the character of the pulse at the time of the first headache, whether it had the globular feeling described by Dr. Gregg? The headache which preceded the convulsions, I mean. It has been my experience that when on the second or third day such a pulse and headache occurs, it is almost a characteristic indication for Belladonna. I think that probably Bell. would have averted the first convulsion if given as soon as indicated by that pulse.

Dr. Kimball—During the night I frequently took the pulse, and often could not find it at all. She would have a convulsion; then in an hour another one; then one in fifteen minutes; then in forty-five minutes. There would be, in other words, a long interval and then a short one. After the Opium she moaned, instead of being in a stertorous sleep. After the dose of Bell. the intervals began to increase, and by morning she was having two hours between the convulsions.

Dr. Long—There is one part of that paper which impresses me very deeply, and makes me feel sad to know that in such extreme cases I stand entirely alone. I have no Dr. Wesselhoeft or Kimball to help me out. There are many Hahnemannians who have to meet the severe strain of their terrible cases alone, unhelped by the friendly hands of their professional brethren. Some of you who are more fortunate do not realize how awful it may sometimes be to stand before a terrible case of sickness, unhelped save by your God and your materia medica.

